# Editorial: Revitalization and sustainable utilization of neglected and underutilized plant species (NUS) for enhanced human nutrition and agroecological resilience

**DOI:** 10.3389/fnut.2026.1980443

**Published:** 2026-10-19

**Authors:** Mihail Chervenkov, Marija Knez, Teodora Ivanova

**Affiliations:** 1 Faculty of Veterinary Medicine, University of Forestry, Sofia, Bulgaria; 2 Department of Plant and Fungal Diversity and Resources, Institute of Biodiversity and Ecosystem Research, Bulgarian Academy of Sciences, Sofia, Bulgaria; 3 Centre of Research Excellence in Nutrition and Metabolism, Group for Nutrition and Metabolism, Institute for Medical Research, National Institute of Republic of Serbia, University of Belgrade, Belgrade, Serbia

**Keywords:** agrobiodiversity, climate adaptation, nutritional benefits, sustainable agriculture, underutilized plants

Global food systems are under increasing pressure to simultaneously provide healthy diets, safeguard biodiversity, mitigate and adapt to climate change, and ensure sustainable livelihoods (1, 2). Addressing these interconnected challenges requires moving beyond reliance on a limited number of major crops toward more diversified, resilient and resource-efficient food systems (3). Within this context, neglected and underutilized plant species (NUS) have emerged as promising yet largely untapped resources. Many possess exceptional nutritional profiles, adaptation to marginal environments and long histories of cultivation, making them valuable contributors to both human nutrition and agroecological sustainability (4, 5). Yet, despite decades of research documenting their nutritional and environmental benefits, relatively few NUS have achieved widespread integration into modern agricultural systems, food value chains or consumer diets (6). Their continued marginalization reflects a complex interplay of agronomic, socioeconomic, cultural and market barriers rather than limitations of the crops themselves. This Research Topic was established to examine how revitalization and sustainable utilization of NUS can contribute to healthier diets, resilient agricultural systems and biodiversity conservation by bringing together evidence spanning nutrition, food science, consumer behavior and agroecology. Although traditionally framed around cultivated crops, the concept of underutilized food resources can also encompass wild and locally harvested species whose nutritional, cultural and ecological values remain insufficiently recognized. Collectively, the contributions demonstrate that revitalization and sustainable utilization are complementary and mutually reinforcing processes that require coordinated action across the entire food system. The Research Topic begins by revisiting rye, highlighting it as a traditional cereal and a resilient crop whose nutritional composition, bioactive compounds and health-promoting properties justify renewed attention in contemporary food systems (Zadeike et al.). While rye exemplifies renewed appreciation of traditional cereals, the review of peas broadens the perspective to grain legumes by highlighting that nutritional quality, environmental sustainability and economic opportunities can be addressed simultaneously through a single multifunctional crop (Ćujić Nikolić et al.). Besides providing high-quality protein and micronutrients, peas improve soil fertility through biological nitrogen fixation, support sustainable crop rotations and offer opportunities within expanding plant-based food markets. The multifunctionality of legumes is further reinforced by the review on chickpeas, which extends beyond their established role in global food systems to emphasize their nutritional, health and culinary potential while highlighting the reduced environmental footprint of food production (Copperstone et al.). Together, these crop-focused reviews provide compelling evidence that NUS can simultaneously support nutrition, environmental sustainability and agricultural resilience. However, demonstrating their potential is only the first step; translating scientific evidence into dietary practice remains an equally important challenge. This question is addressed by comparing dietary recommendations with actual legume consumption patterns across European countries, revealing discrepancies between recommendations and intake and highlighting opportunities for nutrition policy and public health interventions (Grosch and Gedrich). The observed gap between recommendations and consumption naturally raises the question of why nutritionally valuable crops remain underutilized. Recent analyses of traditionally cultivated cereals and legumes show that crops such as rye, pea, and chickpea, despite their adaptation to changing climatic conditions and recognized nutritional benefits remain marginal because of limited production, insufficient characterization and promotion of local genetic resources, dependence on imported raw materials and limited integration into contemporary food markets (7). These findings reinforce the broader barriers identified throughout this Research Topic. Addressing these challenges, one contribution explores the behavioral and socioeconomic factors influencing purchasing decisions regarding cereals, demonstrating that health awareness, perceived value, and accessibility remain key drivers of consumer adoption (Bhatt et al.). For NUS such as buckwheat, successful revitalization requires overcoming limited consumer familiarity while addressing sensory characteristics that shape food preferences and purchasing decisions (Ranic et al.). These behavioral insights are translated into practice through studies showing how recipe development, sensory evaluation and market-oriented product innovation can improve the acceptance of NUS-based foods, demonstrating that successful revitalization depends on nutritional quality as well as culinary creativity and consumer experience. Innovation, however, extends beyond product development. Sustainable processing technologies, such as green hydrogen-assisted extraction, further increase the value of NUS by enabling environmentally friendly recovery of bioactive compounds for functional foods and nutraceutical ingredients (Uçarer et al.). Although improving nutrition and expanding food applications remain central objectives, the importance of NUS extends well-beyond their contribution to human diets (8, 9). The final contributions demonstrate how these taxa also support biodiversity conservation and the resilience of agricultural systems. The exploration of the nutritional composition and calcium speciation of local edible species traditionally used as wild vegetables on Hainan Island illustrates how indigenous knowledge contributes to conserving biodiversity while sustaining food traditions (Wang et al.). Beyond cultivated species, sustainable utilization of underused food resources depends on the social and environmental contexts shaping access and consumption (Němec et al.). Research in Czechia shows that wild foods have distinct access pathways, with plant-based foods linked mainly to forest exposure and regulated animal foods shaped more by social networks and procurement systems. These findings emphasize that revitalization requires attention not only to biological potential but also to cultural practices, knowledge, accessibility, governance, and consumer engagement. Likewise, the overview of millets in the Bundelkhand region of India broadens the perspective from individual crops to agricultural landscapes, showing how resilient traditional species can strengthen food and nutrition security under increasingly challenging climatic conditions (Khan et al.). Viewed collectively, the contributions reveal several overarching themes. First, NUS should be regarded as multifunctional resources capable of simultaneously supporting nutrition, ecosystem services and rural livelihoods. Second, scientific evidence on nutritional quality alone is insufficient to ensure wider adoption; consumer behavior, market development, food innovation and sustainable processing are equally important. Third, biodiversity conservation, traditional knowledge and climate resilience emerge not as separate objectives but as complementary dimensions of sustainable food systems. Together, these studies demonstrate that successful revitalization depends on integrating research across agronomy, nutrition, food science, ecology and the social sciences. Overall, the Research Topic achieved its objective of presenting an integrated perspective on the revitalization and sustainable utilization of NUS. Rather than treating crop production, nutrition, food technology and consumer sciences independently, the Research Topic illustrates how these disciplines interact to determine whether NUS can successfully transition from neglected resources to mainstream components of sustainable food systems. Future research should continue strengthening interdisciplinary collaborations linking plant breeding, characterization of local genetic resources, food technology, behavioral sciences and policy development. Greater attention should also be devoted to developing sustainable value chains, increasing consumer awareness, supporting market innovation and creating enabling policy environments that recognize the contribution of NUS to climate adaptation, biodiversity conservation and healthy diets. Collectively, this Research Topic emphasizes that NUS are not crops of the past but strategic resources for building resilient, diversified and nutrition-sensitive food systems capable of addressing the interconnected challenges of food security and environmental sustainability.

## References

[1] Food and Agricultural Organization of the United Nations (FAO). The State of the World's Biodiversity for Food and Agriculture. In: Bélanger J Pilling D, editors. Rome: Food and Agriculture Organization of the United Nations (2019). Available online at: https://www.fao.org/3/CA3129EN/ca3129en.pdf (Accessed April 28, 2026).

[2] HLPE. Food Security and Nutrition: building a global narrative towards 2030. In: A report by the High Level Panel of Experts on Food Security and Nutrition of the Committee on World Food Security. Rome: FAO (2020). Available online at: https://openknowledge.fao.org/server/api/core/bitstreams/8357b6eb-8010-4254-814a-1493faaf4a93/content (Accessed April 28, 2026).

[3] IPBES. Thematic Assessment Report on the Interlinkages among Biodiversity, Water, Food and Health of the Intergovernmental Science-Policy Platform on Biodiversity and Ecosystem Services. In: Harrison PA McElwee PD van Huysen TL, editors. Bonn: IPBES Secretariat (2024).

[4] Hunter D Borelli T Beltrame DMO Oliveira CNS Coradin L Wasike VW . The potential of neglected and underutilized species for improving diets and nutrition. Planta. (2019) 250:709–29. doi: 10.1007/s00425-019-03169-4 31025196

[5] Future Smart Food. Rediscovering Hidden Treasures of Neglected and Underutilized Species for Zero Hunger in Asia. Bangkok: Food and Agriculture Organization of the United Nations, Regional Office for Asia and the Pacific (2018).

[6] Talucder MSA Ruba UB Robi MAS. Potentiality of neglected and underutilized species (NUS) as a future resilient food: a systematic review. J Agric Food Res. (2024) 16:101116. doi: 10.1016/j.jafr.2024.101116

[7] Chervenkov M Ivanova T Banciu C Dimitrova D. Food choices and climate change – perspectives and challenges for the “new” old crops of the future. Bulg J Agric Sci. (2024) 30(Suppl 1):53–8. Available online at: https://agriacad.eu/ojs/index.php/bjas/article/view/1075

[8] Bassignana CF Bairati L Bruno V Torri L. Consumers' perceptions and attitudes toward a novel labeling framework for underutilized crops: a cross-national comparison. Front Sustain Food Syst. (2025) 9:1629395. doi: 10.3389/fsufs.2025.1629395

[9] Chervenkov M Ivanova T. Traditionally used wild nitrogen-fixing plants in Bulgaria: the forgotten *Lathyrus tuberosus*. *Phytol Balc*. (2023) 29:1314–41. doi: 10.7546/PhB.29.3.2023.6

